# Two Cases of Chromosome 27 Trisomy in Horses Detected Using Illumina BeadChip Genotyping

**DOI:** 10.3390/ani15131842

**Published:** 2025-06-22

**Authors:** Cliona A. Ryan, Donagh P. Berry, Monika Bugno-Poniewierska, Mary-Kate Burke, Terje Raudsepp, Sonja Egan, Jennifer L. Doyle

**Affiliations:** 1 Teagasc, Moorepark, Fermoy, P61 P302 Co. Cork, Ireland; donagh.berry@teagasc.ie; 2 Department of Animal Reproduction, Anatomy and Genomics, University of Agriculture in Krakow, Mickiewicza 24/28 Av., 30-059 Kraków, Poland; monika.bugno-poniewierska@urk.edu.pl; 3 Department of Veterinary Medicine, School of Science and Computing, SETU, X91 CF21 Co. Waterford, Ireland; mary-kate.burke@setu.ie; 4 Department of Veterinary Integrative Biosciences, Texas A&M University, College Station, TX 77840, USA; traudsepp@cvm.tamu.edu; 5 Horse Sport Ireland, Beech House, Millennium Park, Naas, W91 TK7N Co. Kildare, Ireland; segan@horsesportireland.ie (S.E.); jdoyle@horsesportireland.ie (J.L.D.)

**Keywords:** SNP, trisomy, aneuploidy, infertility, horse, FISH, congenital abnormalities, neurologic disorders, Down syndrome, karyotyping

## Abstract

Autosomal trisomy, a genetic disorder characterized by the presence of an extra autosome, is a rare but important chromosomal abnormality in horses, often associated with infertility, developmental abnormalities, and reduced life expectancy. This study represents the largest population-level screening for autosomal trisomy in horses; the analysis used single nucleotide polymorphism (SNP) panel genotype intensity data from 17,078 horses, 6601 of which were juveniles (i.e., ≤12 months of age) when genotyped. Using methodologies adapted from similar screening studies in cattle, the only aneuploidy detected was trisomy 27 in two juvenile male Irish Sport Horses (ISH) (0.03% prevalence among juveniles or 0.01% prevalence in the overall population). One ISH colt was cytogenetically confirmed and displayed no overt external phenotypic abnormalities, while cytogenetics was not undertaken on the other ISH colt, nor was it phenotypically assessed. Parentage analysis revealed that one ISH colt inherited two different copies of chr27 from the sire, demonstrating heterodisomy, likely due to a nondisjunction event during meiosis I in the sire. The other ISH colt inherited two different copies of chr27 from the dam, also indicating heterodisomy; the dam was 23 years of age when the colt was born. Based on the observed prevalence of autosomal trisomy, it can be estimated that at least 3 foals per 10,000 live births are likely to have autosomal trisomy. Though, given that only 74 (i.e., 0.004%) of horses were genotyped within a month of birth, this is likely an underestimate. The economic consequence of undiagnosed trisomy in high-value breeding horses that are potentially infertile could be substantial. As horse genotyping for parentage verification and discovery is transitioning to medium-density single nucleotide polymorphism panels, routine genomic screening for autosomal aneuploidy could be readily undertaken and potentially should form a standard screening prerequisite along with other genetic defects at horse sales. Currently, thoroughbred horses registered for racing are not genotyped, and only a limited number of sport horse studbooks are using SNP genotyping. This highlights an opportunity for those already genotyping to expand their support for breeders through low-cost, high-value chromosomal screening at the time of registration rather than incurring additional costs over the horse’s life cycle to determine the root cause of certain phenotypes owing to the undiagnosed trisomy.

## Simple Summary

The presence of an extra autosome (i.e., autosomal trisomy) is rare in equine populations and is often associated with infertility, developmental anomalies, or early mortality. However, not all trisomies manifest with overt phenotypic abnormalities, making diagnosis challenging without specialized lab testing (i.e., cytogenetics) or molecular analysis. Using single nucleotide polymorphism genotype intensity data from 17,078 horses, two Irish Sport Horse colts were diagnosed with trisomy on chromosome 27, representing a prevalence of 0.03% in a juvenile population. One colt showed no external abnormalities and would likely have gone undiagnosed without genetic testing. As SNP genotyping becomes more common in sport horse registries, it offers a cost-effective opportunity to screen for chromosomal abnormalities early in life, potentially reducing future issues for breeders and owners.

## 1. Introduction

Chromosomal abnormalities in horses are among the most common non-infectious causes of infertility and developmental abnormalities [1]. Chromosomal abnormalities account for 60 to 70% of early pregnancy loss in horses [2] and 30% of fertility or developmental issues in horses [1]. Of these chromosomal abnormalities, aneuploidy, which is a genetic condition characterized by a missing (monosomy) or an extra (trisomy) chromosome [3], often results in non-viable embryos or early abortions in horses [4].

While autosomal monosomy is lethal in most species, trisomy of certain autosomes can result in live births; examples include trisomy 21 in humans (i.e., Down syndrome) [4,5] and trisomy of smaller autosomes in cattle [6]. Only 13 live-born non-mosaic cases of autosomal trisomies have been documented in horses, involving chromosomes 23, 26, 27, 28, 30, or 31 [1,7,8]. Most of these 13 horses exhibited behavioral, neurological, or musculoskeletal issues, with 2 horses requiring euthanasia due to severe health complications. Even where horses with autosomal trisomy have survived, they are generally infertile [1]. The majority of male horses with autosomal trisomy are azoospermic or oligospermic [9,10], caused by arrested spermatogenesis at meiosis [11].

Previous reports of autosomal trisomy in horses have mostly been limited to individual case reports, which are likely biased toward those with severe observable abnormalities. It is possible that horses with autosomal trisomy may exist with mild or no noticeable external phenotypic abnormalities, making them less likely to be identified and documented in case studies. Consequently, horses with undetected autosomal trisomy might be retained as candidates for breeding, which, if infertile, is futile. The only study in horses beyond individual case reports of autosomal aneuploidy was conducted by Bugno et al. [12], who undertook cytogenetic testing of 500 juvenile horses (<3 years old) randomly selected from private studs and farms across Poland to estimate the prevalence of chromosomal abnormalities. However, no cases of non-mosaic autosomal aneuploidy were detected, potentially due to the limited sample size. As a result, reliable estimates of the prevalence of autosomal aneuploidy in live-born horses are not currently available, nor is it known whether horses can carry autosomal trisomy without any obvious (external) phenotypic abnormalities.

Larger-scale cytogenetic screening, like the one undertaken by Bugno, Słota, and Kościelny [12] in horses, is laborious, costly, and impractical. An alternative is low-cost and high-throughput karyotype screening by SNP chip genotyping data, which has been successful in cattle [6,13,14], sheep [15], and humans [16,17,18]; such studies have also been undertaken in cattle embryos, chicken, and salmon [19]. The proposed approach uses SNP chip genotype intensity information to detect chromosomal duplications and deletions. Given that many horse populations, including those in Ireland, are now transitioning to routine SNP genotyping of animals for circa 60,000 SNPs for parentage, this resource offers an opportunity to apply screening algorithms developed in other species to help estimate the prevalence of karyotype abnormalities in the Irish horse population. The objective of the present study was to use available genome-wide medium-density SNP genotypes from Irish horses to estimate the prevalence of autosomal aneuploidy in Ireland.

## 2. Materials and Methods

### 2.1. Genotype Data

Single nucleotide polymorphism genotype intensity data from the Illumina Equine80select chip (Illumina, San Diego, CA, USA) were available for 17,053 horses, all of which had a call rate ≥90%; the population comprised 12,561 horses recorded as females and 4492 recorded as males. Of these, 60% were Irish Sport Horses, 18% were Irish Draughts, and 6% were from foreign studbooks; Thoroughbreds, Irish cobs, Irish sports ponies, Kerry bog ponies, Connemara pony, and horses with no associated studbook each consisted of <5% of the population. Of the 17,053 horses, 6601 (38%) were genotyped when younger than 1 year of age (juveniles), and of these, 74 (i.e., 0.004%) were genotyped within 1 month of birth. The SNP genotype panel consists of 71,248 autosomal and 3511 X-chromosome SNPs. Sex-chromosomal aneuploidy could not be accurately determined in the present study given that no Y SNP probes exist on the Illumina Equine80select chip. Consequently, the genotype of XXY horses would be indistinguishable from that of XX females. Similarly, it is not possible to discriminate between XY males, XO females, SRY-positive and SRY-negative XY females, XX females with X uniparental disomy, or XX females with extremely homozygous X chromosomes due to inbreeding. Therefore, only autosomal SNPs with a locus call rate ≥90% were retained. Following edits, 67,728 autosomal SNPs remained.

### 2.2. Genotype Intensity Data

The B-allele frequency (BAF), the log R ratio (LRR), and R-values of SNP genotype data were used to identify horses that had autosomal aneuploidy as described in detail by Ryan et al. [6] for Thermo Scientific SNP array genotypes in cattle. The R-value is defined as the sum of the raw signal intensity values from both the X and Y fluorescent dyes associated with the reference and alternate alleles for each SNP [20]. The LRR is the log ratio of the observed R-value to the expected R-value, relative to a reference sample [20,21], and is calculated as

(1) $$LRR={log}_{2\;}\left(\frac{X_{sample}+Y_{sample}}{X_{reference}+Y_{reference}}\right)$$

where $X_{sample}$ and $Y_{sample}$ are the respective X and Y signal intensities for the sample of interest and $X_{reference}$ and $Y_{reference}$ are the respective X and Y signal intensities for the reference sample on the panel. An LRR of 0 indicates a neutral copy number, a positive LRR suggests a copy number gain, and a negative LRR indicates a copy number loss [22].

The BAF is calculated by dividing the fluorescence intensity of the B allele by the total fluorescence intensity of that SNP [21]. In the called genotype file generated during genotyping, alleles are designated as A or B. When visualizing a set of consecutive SNPs for a diploid individual in a BAF whole-genome Manhattan-type plot, data typically appear as horizontal bands at 0 (AA), 0.5 (AB), and 1 (BB).

As documented by Ryan, Purfield, Matthews, Rathje, Valldecabres, and Berry [6] and Ryan, Purfield, Matthews, Canedo-Ribeiro, Valldecabres, and Berry [13] for detecting aneuploidy from SNP genotype array data, where chromosomal duplication exists, the heterozygous BAF band typically shifts from 0.5 to approximately 0.33 (AAB) and/or 0.67 (ABB), positioned between the homozygous BAF bands at 0 (AAA) and 1 (BBB). Furthermore, the LRR and R-values associated with duplications are higher than those observed on a standard diploid chromosome. Conversely, in cases of chromosomal deletion, the BAF is restricted to 0 (A) and 1 (B), accompanied by lower LRR and R-values.

### 2.3. Detecting Aneuploidy

Using the BAF, the LRR, and the R-values, the approach proposed by Ryan, Purfield, Matthews, Rathje, Valldecabres, and Berry [6] for detecting autosomal aneuploidy in cattle was used to detect possible cases of autosomal monosomy or trisomy in the sample population. In brief, for each horse, the mean LRR and R-values for the autosome under investigation was expressed relative to the mean and standard deviation of LRR and R-values across all other autosomes (excluding the chromosome being investigated). The population mean and standard deviation of these standardized LRR and R-values were then calculated for each autosome. Additionally, the percentage of SNPs on the autosome under investigation that had a BAF range within the expected heterozygous BAF range of 0.45 to 0.55 (for a diploid genome) was calculated per autosome for each horse.

For each autosome separately, horses with ≤3% of SNPs in the 0.45 to 0.55 BAF range were flagged; this was the approach proposed by Ryan, Purfield, Matthews, Rathje, Valldecabres, and Berry [6] for detecting autosomal aneuploidy in cattle. From these flagged horses, those with standardized LRR and R-values that deviated both by more than 2.5 standard deviations from the respective population mean for that autosome were classified as potentially having autosomal aneuploidy. Horses classified with suspected autosomal monosomy exhibited standardized LRR and R-values that were more than 2.5 standard deviations below the mean, while horses classified with suspected autosomal trisomy had values greater than 2.5 standard deviations above the mean. The genotypes of suspected cases of autosomal aneuploidy were further examined visually by plotting Manhattan plots of LRR and BAF values per SNP across the entire genome to confirm the chromosomal anomaly.

In cases of suspected trisomy, the parent of origin of the extra chromosome was determined by evaluating SNPs on the affected autosome where the sire and dam were homozygous for opposite alleles (i.e., AA in one parent and BB in the other). These informative SNPs with opposing parental homozygotes were used to infer the origin of the third allele by comparing the genotype of the offspring to that of the parents. Offspring genotypes initially called as heterozygous (AB) were recoded based on BAF values; SNP with a BAF around 0.33 were classified as AAB, and SNPs with a BAF around 0.67 as were classified ABB. For each SNP, the parent whose homozygous genotype matched two of the three alleles in the offspring was assumed to have contributed two alleles. These contributions were summed across all informative SNPs to calculate the proportion of alleles inherited from each parent. If one parent consistently contributed two of the three alleles across the chromosome, it was concluded that the trisomy originated from that parent.

In order to determine whether the trisomy arose through isodisomy or heterodisomy, only SNPs on the affected chromosome where the parent identified as the source of the extra chromosome was heterozygous (AB) were analyzed. At these loci, the trisomic offspring must have inherited two alleles from that parent, i.e., the offspring must have inherited either both alleles (A and B), consistent with heterodisomy, or two copies of the same allele (i.e., AA or BB), consistent with isodisomy. If the same allele were inherited twice, a higher proportion of homozygous genotypes (AAA or BBB) would be expected in the offspring, indicating isodisomy. Conversely, inheritance of both parental alleles (A and B) would result in a predominance of heterozygous genotypes (AAB or ABB) in the offspring, indicating heterodisomy.

Furthermore, the owners of horses identified as possibly having autosomal aneuploidy were contacted and asked to provide consent for a veterinary examination by an equine specialist, with the aim of assessing the horses for any external phenotypic abnormalities, developmental issues, or clinical signs that might be associated with chromosomal aneuploidy. During the examination, ethylenediaminetetraacetic acid (EDTA) and serum blood samples were collected. These samples were submitted for hematology and biochemistry analysis. The hematology analysis included the assessment of red blood cell parameters, platelet count, and white blood cell parameters. The red blood cell parameters were red blood cell count, packed cell volume, hemoglobin levels, mean corpuscular volume (MCV), mean corpuscular hemoglobin concentration (MCHC), and mean corpuscular hemoglobin (MCH). The white blood cell tests included total white blood cell count and a differential count of neutrophils, lymphocytes, monocytes, and eosinophils. Biochemistry analysis encompassed protein tests (including total protein, albumin, and globulin levels), liver and muscle enzyme levels, electrolyte levels, iron levels, and kidney function tests.

### 2.4. Karyotyping

With the owner’s consent, cytogenetic analysis was conducted at the University of Agriculture in Krakow on one Irish Sport Horse (ISH) colt suspected of having trisomy of chromosome 27 (chr27) based on the SNP genotyping data. No horses with monosomy were detected in the present study. Blood samples were obtained from the coccygeal vessels using 10 mL lithium heparin evacuated tubes (BD Vacutainer, LH 102 I.U.; BD, Plymouth, UK). To prepare the blood samples for karyotype analysis, heparinised blood was cultured in RPMI 1640 medium with l-glutamine, 10% (*v*/*v*) fetal calf serum (FCS) (Sigma, Deisenhofen, Germany), penicillin and streptomycin sulfate salt (100 g/mL) (Sigma, Deisenhofen, Germany), and lectin from pokeweed (Phytolacca americana; 5 g/mL) (Sigma, St. Louis, MO, USA) for 72 h at 37 °C. Cell division was arrested by the addition of colcemid (Gibco, Thermo Fisher Scientific, Waltham, MA, USA) at a concentration of 10 µg/mL for 120 min, followed by hypotonic treatment using 75 mM potassium chloride. A mixture of methanol and acetic acid in a 3:1 ratio was added on top of the hypotonic solution, and the tubes were inverted for flash fixation.

Metaphases for karyotyping were stained with GTG banding [23]. Images were captured with an Axio Imager M2 microscope equipped with a cooled charge-coupled device camera and the MetaSystem software (MetaSystems, Altlussheim, Germany). A total of 20 metaphase images were captured, and 10 metaphases were karyotyped with Meta System—Neon software (MetaSystem, Germany). The chromosomes were arranged following the International System for Cytogenetic Nomenclature of the Domestic Horse (ISCNH 1997) [24].

### 2.5. Fluorescence In Situ Hybridization (FISH)

FISH was conducted on metaphase spreads using two digoxigenin-labeled horse bacterial artificial chromosome (BAC) clones containing the *MAC16* and *PDGFRL* genes to specifically identify and confirm the presence of an additional copy of chromosome 27 in the metaphase spread. The *MAC16* BAC was labeled with digoxigenin and detected using an anti-digoxigenin–rhodamine conjugate (red signal), while the *PDGFRL* BAC was labeled with Alexa Fluor 488 (green signal). Probe labeling, probe and chromosome denaturation, and hybridization and post-hybridization washes were conducted according to standard procedures described elsewhere [25,26]. Hybridization signals were detected with anti-digoxigenin–rhodamine conjugate (Vector Laboratories), and the results were analyzed under a Axio Imager.D2 fluorescence microscope (Carl Zeiss, Oberkochen, Germany) equipped with a camera (AxioCam MRm; Carl Zeiss) and ZEN software.

## 3. Results

**Figure 1:**
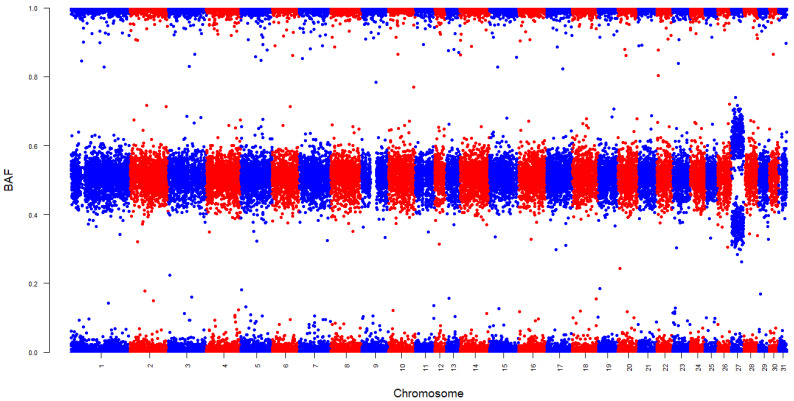

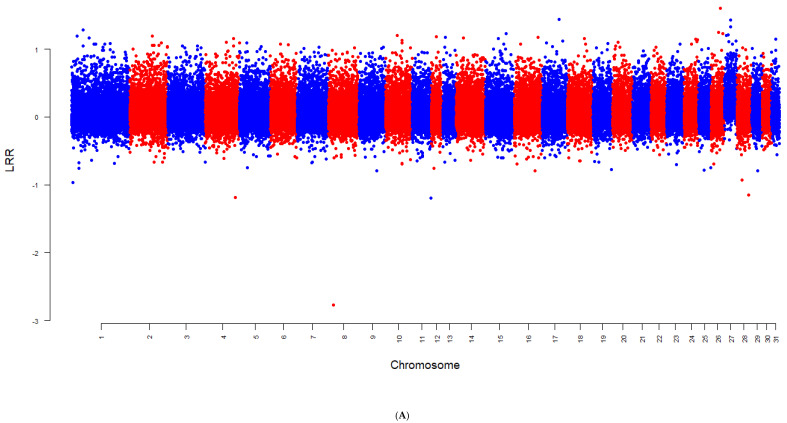

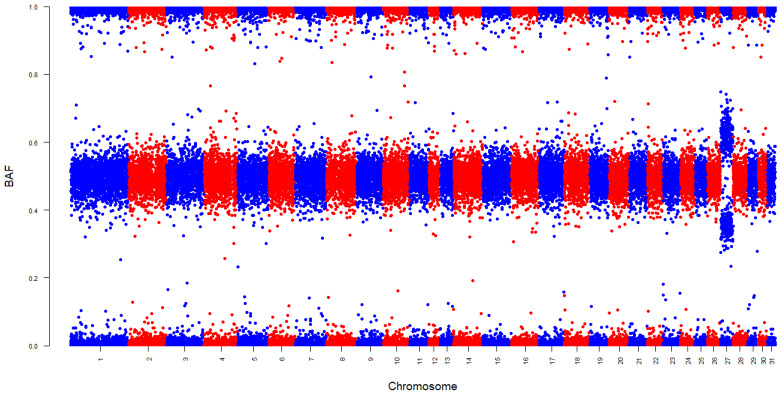

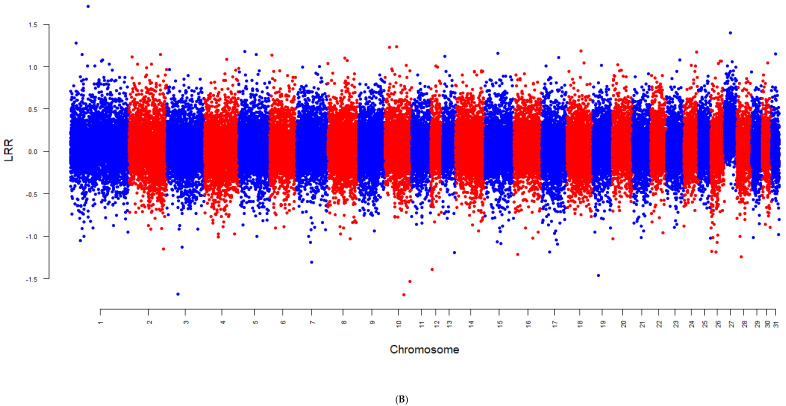
The B-allele frequency (BAF) and log R ratio (LRR) and plots for the (**A**) Thoroughbred x Cob colt and the (**B**) Irish Sport Horse colt diagnosed with trisomy on chr27.

Of the population of 17,078 horses included in this study, only 2 horses, both males, were diagnosed with autosomal trisomy. Both horses were genotyped when they were less than 1 year old, resulting in a prevalence of 0.03% of aneuploidy in the juvenile population. Based on SNP genotyping data, both males had trisomy chr27, which was the only autosomal aneuploidy detected. Both colts were Irish Sport Horses, which belong to an open studbook. One colt was a Cob xThoroughbred cross, referred to as ISH1 from here on out, while the other colt was a Dutch Warmblood x Irish Sport Horse cross, referred to as ISH2 from here on out. Whole-genome BAF and LRR Manhattan plots for the two horses demonstrated clear signatures of trisomy chr27 (Figure 1). Specifically, the BAF plot for both individuals revealed four clusters at 0 (AAA), 0.33 (AAB), 0.67 (ABB), and 1 (BBB) on chr27, whereas the diploid autosomes from these individuals had three clear clusters of BAF at values close to 0 (AA), 0.5 (AB), and 1 (BB). Likewise, the LRR values per SNP were higher on chr27 relative to the diploid autosomes of these two horses.

**Figure 2:**
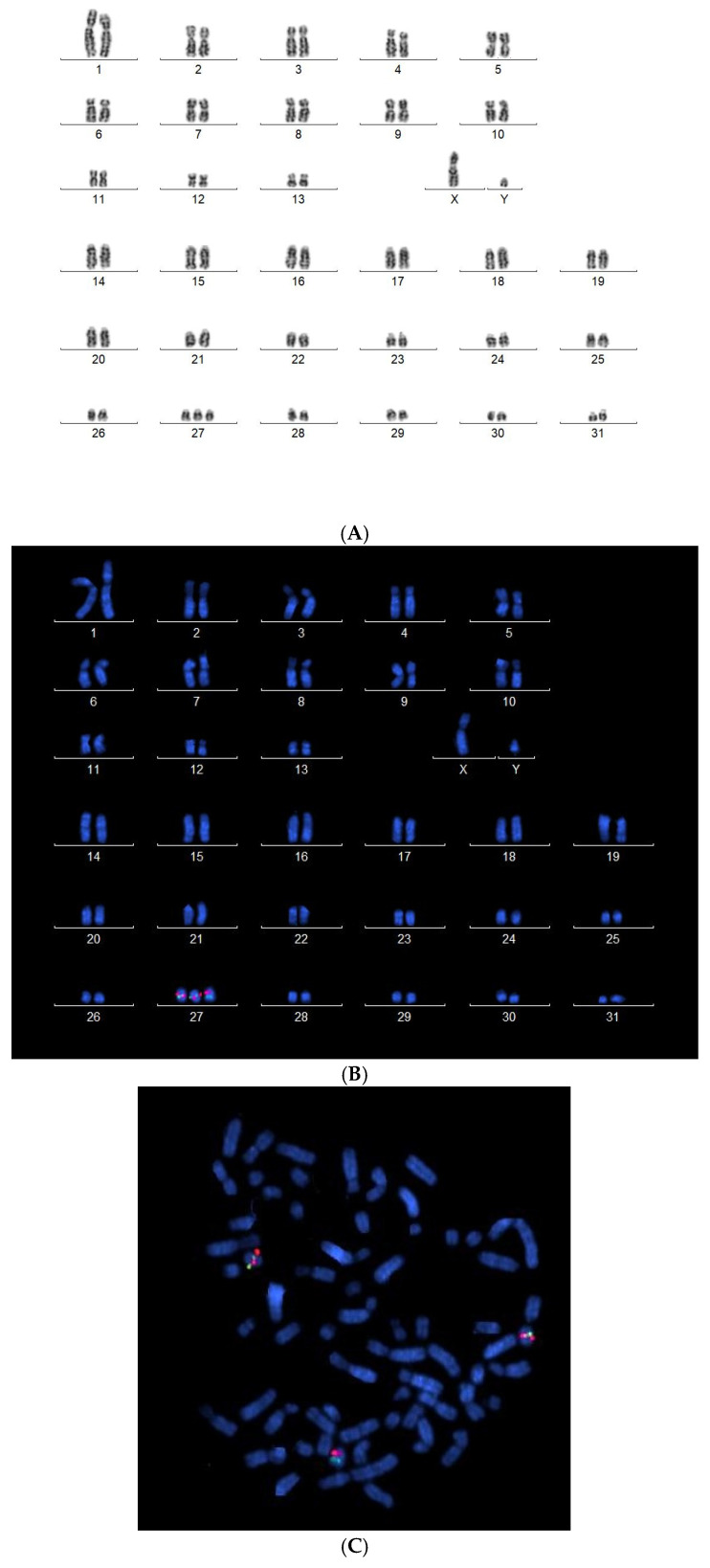
(**A**) G-banded karyotype of the yearling colt with 2n = 65, showing chr27 trisomy, (**B**) DAPI-labeled karyotype following FISH using BAC chromosome 27-specific probes, confirming trisomy of chromosome 27 (The BAC probes: red (rhodamine)-labeled MAC16 and green (streptavidin)-labeled PDGFRL), (**C**) Metaphase spread used to generate the DAPI-labeled karyotype.

The owner of the ISH1 colt with trisomy consented to a veterinary examination of the yearling, whereas the owner of the ISH2 yearling colt declined it. Karyotyping of the ISH1 colt confirmed aneuploidy, showing that the colt had abnormal chromosome number 65, XY with an extra-small acrocentric autosome (Figure 2A). FISH analysis with chr27-specific probe unambiguously identified the extra autosome as chr27 (Figure 2B). Given that at least 100 metaphases were examined by karyotyping and FISH and all demonstrated trisomy chr27 (Figure 2C), mosaicism of ≥3% on chr27 can be ruled out with 99% confidence, or mosaicism of ≥2% can be ruled out with 95% confidence [27].

### 3.1. Parental Origin

Parental origin of the extra copy of chromosome 27 was established for both colts by analyzing SNPs at which the sire and dam were homozygous for opposite alleles (i.e., AA in one parent and BB in the other) (Supplementary Table S1). A total of 87 such SNPs were identified for the ISH1 colt and 102 for the ISH2 colt. The ISH1 colt inherited the extra copy of chr27 from its sire (Table 1), who was 6 years old when the colt was born. The sire was in the youngest 15% of stallions used in Ireland in 2023 [28]. The sire has 37 other progeny, with genotypes available for 11 of them, none of which exhibited any autosomal aneuploidy. The ISH2 colt inherited the extra chr27 from its dam (Table 1), who was 23 years old when the colt was born. The dam was in the oldest 5% of dams that had a foal in Ireland in 2023 [28]. The dam had nine other progeny, with the Irish Sport Horse colt being her most recent; no genotypes were available for the other nine progeny.

To determine whether the trisomy arose through isodisomy or heterodisomy, SNPs on chromosome 27 were further evaluated where the parent identified as the source of the extra chromosome was heterozygous (AB genotype). For the ISH2 colt, 262 SNPs were analyzed where the dam was heterozygous. Of these, 242 showed heterozygous genotypes (AAB or ABB), and 20 were homozygous (AAA or BBB). For the ISH1 colt, 240 SNPs were analyzed where the sire was heterozygous, and the ISH1 colt was heterozygous (AAB/ABB) across all 240 SNPs. These results indicate that both colts inherited two different alleles from the parent of origin, consistent with heterodisomy (Table 2).

### 3.2. Phenotypic Assessment

**Figure 3:**
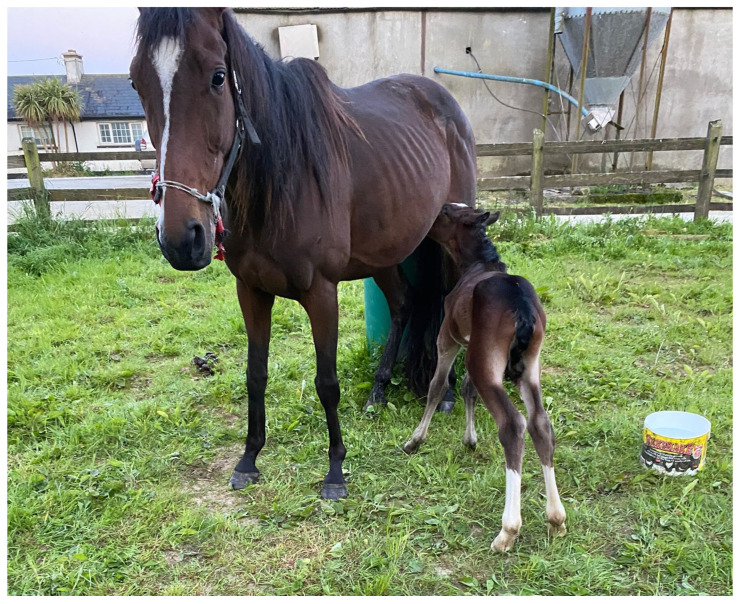
Photograph of the ISH1 colt with chr27 trisomy, shown here as a foal. The image illustrates mild bilateral forelimb flexor tendon hyperflexion (commonly referred to as “contracted tendons”) and mild bilateral hindlimb flexor tendon laxity.

The owner of the ISH1 colt provided background information on the colt. The owner reported no issues with gestation and parturition. The owner reported that the yearling colt, however, displayed clinical signs consistent with neonatal maladjustment syndrome (i.e., dummy foal syndrome) during his first week of life, for which he was treated with intravenous fluids and the Madigan squeeze technique. Four photographs taken by the owner of the back and the side of the colt when he was approximately 1 week of age were analyzed by the veterinarian and indicated mild bilateral forelimb flexor tendon hyperflexion (i.e., contracted tendons) and mild bilateral hindlimb flexor tendon laxity (Figure 3).

Apart from being born with neonatal maladjustment syndrome, the owner described the colt as relatively normal but reported that the yearling colt displayed unusual social behavior when turned out with his paddock mates (other horses of similar age). The yearling colt appeared to be slow to react to intraspecies social cues and appeared to be “pushed around” by or isolated from his paddock mates as a result.

**Figure 4:**
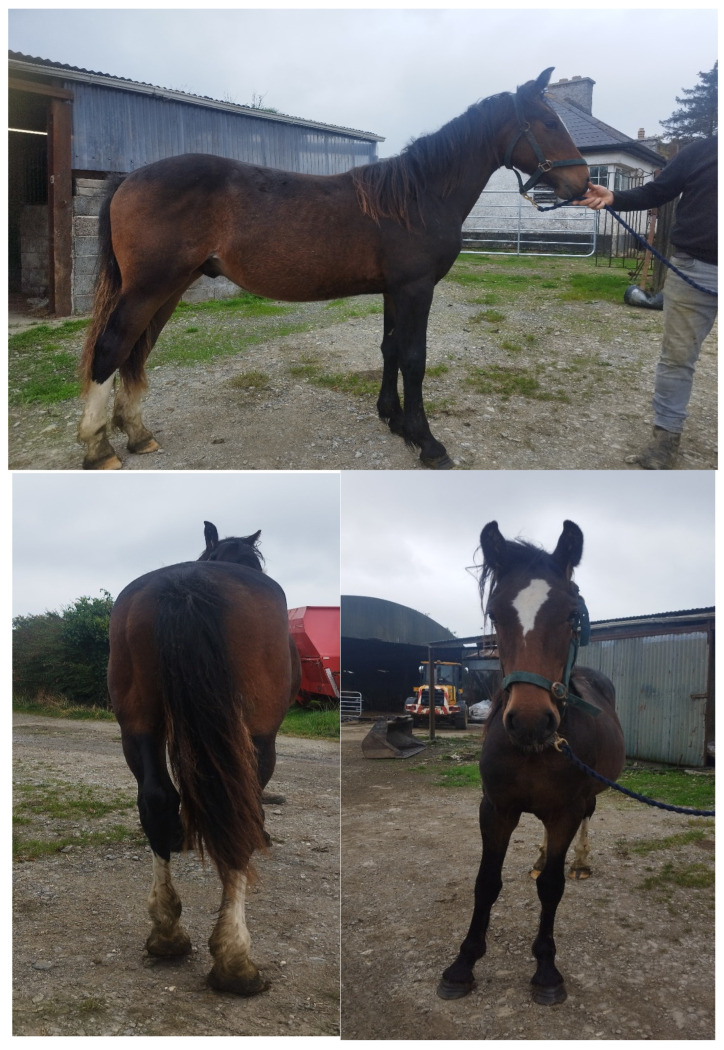
Photographs of the ISH1 yearling colt with chr27 trisomy appearing bright and alert and in overall good health.

**Figure 5:**
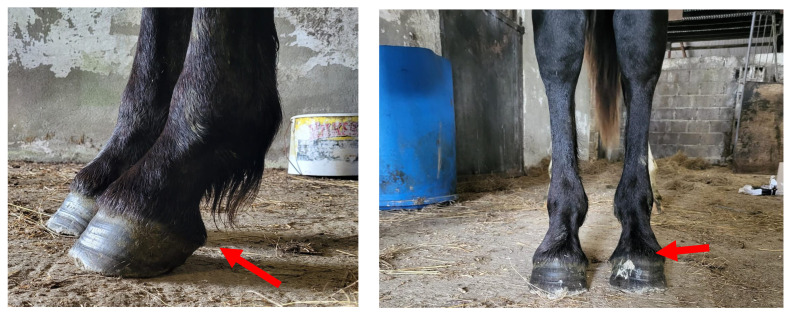
Photograph of the ISH1 colt with chr27 trisomy displaying mild to moderate clubfoot of the left forefoot.

As part of this study, the ISH1 colt was examined externally by a veterinarian on the farm as a yearling. On initial examination, the colt appeared bright, alert, responsive, and in good general health (Figure 4). The colt was smaller in size relative to his two contemporaries within the herd of similar age and breed. No obvious abnormalities of the head, eyes, or teeth were noted on examination. A grade-one holosystolic cardiac murmur was audible at the heart base on the left-hand side. The musculoskeletal system was largely within normal limits, baring a mild to moderate clubfoot appearance of the left forefoot and the straight hock conformation bilaterally (Figure 5).

The penis and prepuce of the ISH1 colt were both normal on visual examination of the reproductive organs. Initially, only the right testicle was palpable within the scrotum. Following sedation with 0.2 mL of detomidine hydrochloride (Domosedan 10 mg/mL solution) and 0.2 mL of butorphanol tartrate (Torbugesic 10 mg/mL solution) intravenously, the left testicle was found via ultrasound to be retained within the left inguinal canal. The left testicle could be palpated within the scrotum following gentle manipulation. Both testicles were examined and measured via ultrasound. The left testicle (3 cm × 5 cm) was smaller in size compared to the right testicle (6 cm × 8 cm), which likely indicates that the left testicle descended more recently than the right. The ultrasonographic appearance of both testicles and associated structures was within normal limits.

Hematology blood analysis revealed that the red blood cell count, packed cell volume, and hemoglobin levels were all below the reference ranges, indicating mild anaemia (Supplementary Table S2), while the eosinophil levels indicated eosinopenia. The biochemistry analysis revealed mild hypoalbuminemia, as both albumin and total protein levels were slightly below normal (Supplementary Table S2). These biochemistry findings are consistent with a high parasitic worm burden and are not thought to be related to the genetic abnormality and are likely normal in the ISH1 paddock mates.

## 4. Discussion

This study represents the most extensive screening effort to date for autosomal aneuploidy in horses, comprising 17,053 horses from seven different breeds. Unlike previous studies, which have primarily focused on individual case reports [7,8,9] or small-scale cytogenetic screening initiatives [12], the present study used a cost-effective SNP-based karyotype screening approach to generate population-level insights into the prevalence of autosomal aneuploidy. By overcoming the limitations of traditional cytogenetic screening, such as high costs and, by extension, limited sample sizes, one conclusion from the present study was that autosomal trisomy can exist in horses even without obvious external phenotypic abnormalities. The scale of this study not only provides the first prevalence estimate of autosomal aneuploidy in horses but also underscores the utility of SNP-based screening as a valuable tool for large-scale detection of chromosomal duplications and deletions in equine populations. This is especially true in populations that have already embarked on SNP-panel-based genotyping strategies, adding to the range of information that can be generated from such data [29], providing an opportunity for annual foal surveillance, which could support education for breeders and vets.

### 4.1. Prevalence of Aneuploidy

No cases of autosomal monosomy were detected in the present study, which is expected given autosomal monosomy is generally lethal and has not been detected in live-born livestock animals [30]. Nonetheless, autosomal monosomy has been recorded in early equine pregnancy losses [31]. The detection of two cases of autosomal trisomy in a population of 17,078 genotyped horses corresponds to a prevalence of 0.01%. Notably, 6601 juvenile horses were genotyped, yielding a prevalence of 0.03% in juveniles. This prevalence is consistent with reported prevalence rates in cattle, where 0.01% of 779,138 juvenile cattle genotyped also on a SNP-genotype array were diagnosed with autosomal trisomy [6].

In the present study, trisomy was detected only for chr27, with no evidence of trisomy of other autosomes. In a population of 779,138 cattle, Ryan, Purfield, Matthews, Rathje, Valldecabres, and Berry [6] reported trisomy on 10 different autosomes. However, the cattle dataset used by Ryan, Purfield, Matthews, Rathje, Valldecabres, and Berry [6] was approximately 47 times larger than the dataset used in the present study, thus providing greater power to detect rare events such as autosomal trisomy. Based on the prevalence rates of trisomy observed in cattle and adjusting for the smaller sample size of the horse dataset, the likelihood of detecting trisomy on horse autosomes in the current population is exceedingly low. For instance, the prevalence of the rarest trisomy detected in juvenile cattle was that of chr6 at 0.00013% [6]. Given this prevalence, almost 800,000 genotyped horses would be required to detect one case, which equates to fewer than one case (approximately 0.02 cases) expected in a population of 17,000 horses.

An additional feature of the structure of the present study in relation to detecting aneuploidy was that horses are genotyped at any stage of their life so the dataset used in the current study likely does not include genotypes from horses that would have died shortly after birth, potentially due to trisomy of larger autosomes. While trisomy on only chr27 was detected in the present study, previous studies in horses have reported trisomy of horse chr23, 26, 28, 30, and 31 [1,7,8], suggesting additional cases of autosomal trisomy may exist but were undetected here. This may be because, unlike calves, which are genotyped at birth in Ireland, horses in this study were rarely genotyped soon after birth; only 74 of the 17,053 horses (0.004%) were genotyped within a month of being born. Furthermore, foals with external phenotypic abnormalities may not have been genotyped by their owners. While aneuploidy can occur on any chromosome [32,33], the viability of the embryo often depends on which chromosome is affected. Specifically, trisomy on longer autosomes are more likely to be aborted before birth [6,31,34]. If horses with trisomy on larger autosomes died within the first two weeks of life, as observed in cattle [6], they would likely not be genotyped. For example, the stated requirement to have to genotype almost 800,000 animals to detect a single case with a prevalence of 0.00013% (chr6 in Ryan et al. [6]) is compounded by the fact that these 800,000 foals would have to be genotyped at birth; [6] reported that all 19 cattle cases of trisomy of chr12, 15, 20, and 24 died within 15 days of birth. Consequently, the prevalence estimates in the present study could be an underestimate of the true prevalence of autosomal trisomy in horses.

While the study provides a robust estimate of autosomal aneuploidy prevalence in horses, it does not account for sex-chromosomal aneuploidies. This limitation arises from the absence of Y-chromosome SNP probes on the genotyping chip, which prevents reliable detection of abnormalities such as XXY (Klinefelter-like), XO (Turner-like), and SRY-negative XY females.

### 4.2. Parental Origin of Additional Chromosome

Aneuploidy is often attributed to meiotic errors, and in humans, the risk increases with advancing maternal age [33]. From a study of 782 humans with Down syndrome (trisomy chr21), the extra chromosome originated from the mother in over 90% of the cases [32]. Of those that inherited the extra chromosome from the mother, 70% of the errors originated during maternal meiosis I. Similarly, of the 134 cattle with trisomy and genotyped parents, the extra autosome originated from the dam in 92% of trisomy cases [6]. While the ISH1 colt in the current study inherited the extra chromosome from his sire, the ISH2 colt inherited the extra chromosome from his dam. Both cases were heterodisomy, which usually occurs due to a nondisjunction error during meiosis I, where homologous chromosomes fail to separate, and both are passed to the offspring. The parental origin of trisomy in horses has been previously reported in only one case, where the extra chromosome was confirmed to originate from the dam [35]. In another case, it was presumed (but not proven) to be maternally inherited due to the dam’s advanced maternal age (26 years) [36].

Just as the risk of aneuploidy increases with maternal age in humans [33], the same may be true for horses; of the 10 live-born foals previously reported in other studies to have autosomal trisomy where the dam’s age was reported, 5 of the dams were aged between 14 and 28 years when they gave birth to the foal with trisomy [8,10,36,37,38]. Only 1 of the 10 dams was <5 years old when she gave birth to the foal with trisomy [8]. Similarly, the dam of the ISH2 colt in the present study, identified as the parent of origin for the extra chromosome, was 23 years old when the colt was born. The increasing risk with maternal age in horses is not unexpected given that mare oocytes are subject to age-related aneuploidy [39]. In contrast, the sire of the ISH1 colt identified as the parent of origin of the extra chromosome was only 6 years old when the colt was born.

### 4.3. Chromosome-Specific Aneuploidy

The lethality of autosomal aneuploidy is influenced by the imbalance effects of several major genes together with many minor genes specific to the individual chromosome [40]. As a result, the prevalence of trisomy may be similar for each chromosome at the time of conception but may differ greatly among abortuses and live-borns [41]. For instance, while trisomy of all autosomes, with the exception of chr7, has been observed in cattle embryos [32], trisomy of only 10 chromosomes has been detected in a very large population of live-born cattle [6]. Similarly, trisomy on chr1 and chr11 in humans has been reported in multiple spontaneous abortions but never reported in live-borns, suggesting lethality in each instance [41]. To the best of our knowledge, no large-scale screening study has been conducted on the prevalence of aneuploidy in equine embryos. However, Shilton, Kahler, Davis, Crabtree, Crowhurst, McGladdery, Wathes, Raudsepp, and de Mestre [31] reported that, of 256 equine products of conception analyzed from early pregnancy losses (i.e., embryos lost before 55 days of gestation), triploidy was identified in 42% of cases, while trisomy and monosomy were detected in 4.6% and 4.2% of the products of conception, respectively.

Trisomy of chr27 appears to be the most common autosomal trisomy in both live-born cattle [6] and horses (the present study). Interestingly, both species share conserved synteny with human chromosomes 8p and 4q [42]. The relatively high prevalence of trisomy chr27 in live-born cattle and horses may be linked to the small size and low gene density of chr27 in cattle and chr27 in horses compared to other autosomes in their respective genomes (Supplementary Tables S3 and S4). For instance, chr27 in horses has a total gene density of only 8.4 genes per Mb, whereas chr12, a more gene-dense autosome, has a total of 24.7 genes per Mb (Supplementary Table S4). Similarly, in cattle, chr27 has a total gene density of 8.3 genes per Mb, compared to 25 genes per Mb on chr18 (Supplementary Table S3). These findings support the general observation that aneuploidy of smaller, gene-poor autosomes tends to be less detrimental [43].

A similar pattern is observed in humans; while aneuploidy can occur on all autosomes, only trisomy on chromosomes 13, 18, and 21 are viable at birth [41]. These three chromosomes are the gene-poorest autosomes in the human genome [4]. The relationship between chromosome size and the viability of aneuploid embryos appears to hold true in horses as well. All reported cases of live-born, non-mosaic autosomal trisomy in horses involve shorter autosomes, including chr23, 26, 27, 28, 30, and 31 [1,7,8]. Of the 13 documented cases of live-born, non-mosaic autosomal trisomy in horses, 38% were on chr27, with an additional 30% being chr30. Notably, these two chromosomes are among the four autosomes with the lowest total gene count in the equine genome (Supplementary Table S4). However, it is important to note that chr29 in horses is also among the four autosomes with the lowest gene counts in the equine genome, yet no live-born horses with trisomy on chr29 have been reported.

### 4.4. Phenotypic Symptoms

Previous studies documenting autosomal aneuploidy in horses have primarily focused on individual cases trying to resolve the cause of notable phenotypic abnormalities. These case reports have documented a variety of musculoskeletal issues, such as facial asymmetry [7], brachygnathia inferior [36], articulated splint bones [8], and angular limb deformities [8,36]. Neurological impairment has also been documented in some of these cases [35], suggesting that candidates for further investigation often consist of horses with visible abnormalities. In fact, across species, case studies documenting karyotype abnormalities [7,44,45] are generally on animals with some external phenotypic abnormalities.

The yearling ISH1 colt with trisomy in the current study had no discernible external symptoms, albeit he was smaller than his contemporaries on the farm; smaller size is a characteristic reported in other cases of equine autosomal aneuploidy [7,9,10]. Given that this colt exhibited no severe phenotypic abnormalities, he would have likely gone undetected as harboring a karyotype abnormality. Despite his lack of observable symptoms, he is potentially (likely) infertile, as autosomal trisomy in males typically results in azoospermia or oligospermia due to arrested spermatogenesis during meiosis [9,10,11]. Nonetheless, the penis and prepuce of the ISH1 colt with trisomy chr27 were normal on visual examination of the reproductive organs, and the ultrasonographic appearance of testicles and associated structures were within normal limits. Likewise, there were no clear external signs of infertility other than cryptorchidism in the colt with chr27 trisomy documented by Brito, Sertich, Durkin, Chowdhary, Turner, Greene, and McDonnell [9], but no sperm was found during repeated attempts to collect semen. This underscores the importance of population-wide genomic screening for detecting individuals with aneuploidy who, though obviously asymptomatic, may still present reproductive and economic risks.

### 4.5. Reduced Life Expectancy

The colts with chr27 trisomy, as identified in the present study, were 14 and 16 months of age when the analysis for this study was conducted. Survival beyond infancy for horses with autosomal trisomy is thought to be rare, with intensive care sometimes required to ensure survival [46]. To date, only one horse with autosomal trisomy, which was chr26, has been documented to survive beyond 2 years of age [8]. Previous reports of chr27 trisomy in horses highlight its severe health repercussions. For example, a colt with chr27 trisomy was euthanized at 6 months of age due to health complications [37], while a filly with mosaic trisomy of chr27 was euthanized at 2 years of age due to health issues [46]. In stark contrast, the yearling ISH1 colt with trisomy chr27 described in the present study exhibited no obvious health issues. The colt with trisomy chr27 documented by Brito, Sertich, Durkin, Chowdhary, Turner, Greene, and McDonnell [9] was electively euthanized at 2 years of age, but no obvious health ailments were reported that might have impacted survival into adulthood. The only other documented case of trisomy chr27 involved a foal [38], although its lifespan was not reported.

The only autosomal trisomy in humans where the individuals typically survive into adulthood is trisomy chr21 (Down syndrome) [47]. Live-born cattle with autosomal trisomy have been documented to have a markedly reduced life expectancy [6]. In a study of nearly 800,000 genotyped cattle from multiple breeds, including calves genotyped at birth and those that were stillborn, autosomal trisomies involving the larger chromosomes (chrs6, 12, 15, 20, and 24) typically did not survive beyond 2 weeks of age [6]. Notably, the only cattle with autosomal trisomy that survived beyond 24 months were those with trisomy on the shorter autosomes (chrs27, 28, or 29), which typically carry fewer genes [6].

## 5. Conclusions

An approach developed for identifying autosomal aneuploidy in cattle using SNP-panel genotype intensity metrics was applied to 17,078 horses, of which 6601 were genotyped as juveniles. Only two horses, both of which were still juveniles, were detected with autosomal aneuploidy; both had trisomy of chr27, translating to a prevalence of 0.03% in the juvenile population. Although the occurrence rate is low, undiagnosed trisomy can be a nuisance as horses with the condition, such as the colt described in this study, may appear outwardly normal during early life but may potentially fail to meet expectations for performance, fertility, or conformation. The economic implications of undiagnosed trisomy in horses could be substantial, particularly for buyers who may incur a large cost of purchasing a breeding filly or a young colt. To mitigate these risks, routine screening for aneuploidy using SNP-panel genotype information could be considered a standard prerequisite for horse sales, particularly for high-value breeding or performance animals. Moreover, the inability to properly diagnose sex chromosome aneuploidy using the approach in the present study because of a lack of Y-chromosome SNPs points to the need to include Y-chromosome SNPs on genotype panels.

## Figures and Tables

**Table 1:** Determination of the parent of origin of the extra chromosome 27 based on SNPs where the sire and dam were homozygous for opposite alleles (i.e., AA vs. BB).

Colt ID	Number of SNPs with Opposing Parental Homozygotes	% of Alleles Dam	% of Alleles from Sire	Inferred Parent of Origin of Extra Chromosome
ISH1	87	66.3%	33.3%	Extra chromosome inherited from the dam
ISH2	102	33.7%	66.0%	Extra chromosome inherited from the sire

**Table 2:** Determination of heterodisomy versus isodisomy based on SNPs where the parent of origin was heterozygous (AB).

Colt ID	Number of AB SNPs in Parent of Origin	Heterozygous Genotypes in the Colt	Homozygous Genotypes in the Colt	Interpretation
ISH1	240	240	0	Heterodisomy
ISH2	262	242	20	Heterodisomy

## Data Availability

No new data were created or analyzed in this study.

## Supplementary Materials

The following supporting information can be downloaded at: https://www.mdpi.com/article/10.3390/ani15131842/s1. Table S1: SNP-level analysis used to determine parental origin of the extra chromosome in ISH1 and ISH2; Table S2: Hematology and biochemistry test results for the ISH1 colt; Table S3: The length and the number of genes on each bovine autosome according to the ARS-UCD 1.2 genome build; Table S4: The length and the number of genes on each equine autosome according to the EquCab3.0., adapted from Raudsepp et al. [48].

## Abbreviations

The following abbreviations are used in this manuscript:

SNP	Single Nucleotide Polymorphism
ISH	Irish Sport Horse
chr27	Chromosome 27
LRR	Log R Ratio
EDTA	Ethylenediaminetetraacetic Acid
BAC	Bacterial Artificial Chromosome
ISCNH	International System for Cytogenetic Nomenclature of the Horse
