# Supplementary material for: Two Cases of Chromosome 27 Trisomy in Horses Detected Using Illumina BeadChip Genotyping
Source: Animals (Basel). 2025 Jun 22;15(13):1842. doi: 10.3390/ani15131842 (PMC12248643; doi:10.3390/ani15131842)
Supplement: Supplementary file 1 [file animals-15-01842-s001.zip › animals-3630236-supplementary.pdf]

**Supplementary Table S1.** SNP-level analysis used to determine parental origin of the extra chromosome in ISH1 and ISH2.

| Animal ID | SNP | Colts genotype | Dams genotype | Sires genotype | Number of informative SNPs | Number of alleles from dam | Number of alleles from sire | Proportion of alleles inherited from dam | Proportion of alleles inherited from sire |
|---|---|---|---|---|---|---|---|---|---|
| ISH1 | Affx-101276407 | ABB | BB | AA | 261 | 2 | 1 | 66.28 | 33.33 |
| ISH2 | Affx-101397280 | ABB | AA | BB | 306 | 1 | 2 | 33.66 | 66.01 |
| ISH1 | Affx-101413346 | AAB | AA | BB | 261 | 2 | 1 | 66.28 | 33.33 |
| ISH1 | Affx-101443110 | ABB | BB | AA | 261 | 2 | 1 | 66.28 | 33.33 |
| ISH2 | Affx-101483013 | AAB | AA | BB | 306 | 2 | 1 | 33.66 | 66.01 |
| ISH1 | Affx-101696490 | ABB | BB | AA | 261 | 2 | 1 | 66.28 | 33.33 |
| ISH1 | Affx-101720984 | ABB | BB | AA | 261 | 2 | 1 | 66.28 | 33.33 |
| ISH1 | Affx-101736769 | ABB | BB | AA | 261 | 2 | 1 | 66.28 | 33.33 |
| ISH2 | Affx-101835362 | AAB | BB | AA | 306 | 1 | 2 | 33.66 | 66.01 |
| ISH1 | Affx-101836247 | ABB | BB | AA | 261 | 2 | 1 | 66.28 | 33.33 |
| ISH2 | Affx-101912275 | AAB | BB | AA | 306 | 1 | 2 | 33.66 | 66.01 |
| ISH1 | Affx-101930482 | ABB | BB | AA | 261 | 2 | 1 | 66.28 | 33.33 |
| ISH1 | Affx-101941477 | AAB | AA | BB | 261 | 2 | 1 | 66.28 | 33.33 |
| ISH1 | Affx-101942642 | ABB | BB | AA | 261 | 2 | 1 | 66.28 | 33.33 |
| ISH2 | Affx-102135815 | ABB | AA | BB | 306 | 1 | 2 | 33.66 | 66.01 |
| ISH2 | Affx-102177418 | ABB | AA | BB | 306 | 1 | 2 | 33.66 | 66.01 |
| ISH1 | Affx-102196251 | AAB | AA | BB | 261 | 2 | 1 | 66.28 | 33.33 |
| ISH2 | Affx-102444338 | ABB | AA | BB | 306 | 1 | 2 | 33.66 | 66.01 |
| ISH2 | Affx-102473737 | AAB | BB | AA | 306 | 1 | 2 | 33.66 | 66.01 |
| ISH2 | Affx-102505809 | ABB | AA | BB | 306 | 1 | 2 | 33.66 | 66.01 |
| ISH1 | Affx-102505809 | ABB | BB | AA | 261 | 2 | 1 | 66.28 | 33.33 |
| ISH2 | Affx-102626946 | AAB | BB | AA | 306 | 1 | 2 | 33.66 | 66.01 |
| ISH1 | Affx-102818314 | AAB | AA | BB | 261 | 2 | 1 | 66.28 | 33.33 |

| | | | | | | | | | |
|---|---|---|---|---|---|---|---|---|---|
| ISH2 | Affx-102833310 | ABB | AA | BB | 306 | 1 | 2 | 33.66 | 66.01 |
| ISH1 | Affx-102878899 | AAB | AA | BB | 261 | 2 | 1 | 66.28 | 33.33 |
| ISH2 | Affx-102912477 | ABB | AA | BB | 306 | 1 | 2 | 33.66 | 66.01 |
| ISH1 | Affx-103020340 | ABB | BB | AA | 261 | 2 | 1 | 66.28 | 33.33 |
| ISH1 | AX-103050765 | AAB | AA | BB | 261 | 2 | 1 | 66.28 | 33.33 |
| ISH1 | AX-103137188 | AAB | AA | BB | 261 | 2 | 1 | 66.28 | 33.33 |
| ISH1 | AX-103151665 | AAB | AA | BB | 261 | 2 | 1 | 66.28 | 33.33 |
| ISH2 | AX-103158497 | AAB | BB | AA | 306 | 1 | 2 | 33.66 | 66.01 |
| ISH1 | AX-103430664 | AAB | AA | BB | 261 | 2 | 1 | 66.28 | 33.33 |
| ISH1 | AX-103706530 | ABB | BB | AA | 261 | 2 | 1 | 66.28 | 33.33 |
| ISH2 | AX-103724473 | ABB | AA | BB | 306 | 1 | 2 | 33.66 | 66.01 |
| ISH1 | AX-103754556 | AAB | AA | BB | 261 | 2 | 1 | 66.28 | 33.33 |
| ISH2 | AX-104027800 | AAB | BB | AA | 306 | 1 | 2 | 33.66 | 66.01 |
| ISH1 | AX-104027800 | ABB | BB | AA | 261 | 2 | 1 | 66.28 | 33.33 |
| ISH2 | AX-104350261 | ABB | AA | BB | 306 | 1 | 2 | 33.66 | 66.01 |
| ISH2 | AX-104416798 | AAB | BB | AA | 306 | 1 | 2 | 33.66 | 66.01 |
| ISH1 | AX-104637425 | AAB | AA | BB | 261 | 2 | 1 | 66.28 | 33.33 |
| ISH2 | AX-104795135 | ABB | AA | BB | 306 | 1 | 2 | 33.66 | 66.01 |
| ISH1 | BIEC2-700250 | ABB | BB | AA | 261 | 2 | 1 | 66.28 | 33.33 |
| ISH1 | BIEC2-705581 | AAB | AA | BB | 261 | 2 | 1 | 66.28 | 33.33 |
| ISH2 | BIEC2-706880 | ABB | AA | BB | 306 | 1 | 2 | 33.66 | 66.01 |
| ISH1 | BIEC2-708018_ilmndup1 | AAB | AA | BB | 261 | 2 | 1 | 66.28 | 33.33 |
| ISH1 | BIEC2-708019 | ABB | BB | AA | 261 | 2 | 1 | 66.28 | 33.33 |
| ISH2 | BIEC2-709885 | ABB | AA | BB | 306 | 1 | 2 | 33.66 | 66.01 |
| ISH2 | BIEC2-713319 | ABB | AA | BB | 306 | 1 | 2 | 33.66 | 66.01 |
| ISH2 | BIEC2-714304 | AAB | BB | AA | 306 | 1 | 2 | 33.66 | 66.01 |
| ISH2 | BIEC2-715291 | ABB | AA | BB | 306 | 1 | 2 | 33.66 | 66.01 |
| ISH1 | BIEC2-715291 | AAB | AA | BB | 261 | 2 | 1 | 66.28 | 33.33 |
| ISH1 | BIEC2-715469 | ABB | BB | AA | 261 | 2 | 1 | 66.28 | 33.33 |
| ISH1 | BIEC2-715477 | AAB | AA | BB | 261 | 2 | 1 | 66.28 | 33.33 |
| ISH2 | BIEC2-720332 | ABB | AA | BB | 306 | 1 | 2 | 33.66 | 66.01 |

| | | | | | | | | | |
|---|---|---|---|---|---|---|---|---|---|
| ISH2 | BIEC2-720851 | AAB | BB | AA | 306 | 1 | 2 | 33.66 | 66.01 |
| ISH1 | BIEC2-720851 | AAB | AA | BB | 261 | 2 | 1 | 66.28 | 33.33 |
| ISH1 | BIEC2-721307 | AAB | AA | BB | 261 | 2 | 1 | 66.28 | 33.33 |
| ISH1 | BIEC2_700247 | ABB | BB | AA | 261 | 2 | 1 | 66.28 | 33.33 |
| ISH1 | BIEC2_700251 | AAB | AA | BB | 261 | 2 | 1 | 66.28 | 33.33 |
| ISH2 | BIEC2_700268 | ABB | AA | BB | 306 | 1 | 2 | 33.66 | 66.01 |
| ISH1 | BIEC2_700313 | AAB | AA | BB | 261 | 2 | 1 | 66.28 | 33.33 |
| ISH2 | BIEC2_700356 | ABB | AA | BB | 306 | 1 | 2 | 33.66 | 66.01 |
| ISH2 | BIEC2_700396 | AAB | BB | AA | 306 | 1 | 2 | 33.66 | 66.01 |
| ISH1 | BIEC2_700453 | AAB | AA | BB | 261 | 2 | 1 | 66.28 | 33.33 |
| ISH1 | BIEC2_700466 | AAB | AA | BB | 261 | 2 | 1 | 66.28 | 33.33 |
| ISH2 | BIEC2_700474 | ABB | AA | BB | 306 | 1 | 2 | 33.66 | 66.01 |
| ISH1 | BIEC2_700474 | AAB | AA | BB | 261 | 2 | 1 | 66.28 | 33.33 |
| ISH2 | BIEC2_701156 | AAB | BB | AA | 306 | 1 | 2 | 33.66 | 66.01 |
| ISH1 | BIEC2_704094 | ABB | BB | AA | 261 | 2 | 1 | 66.28 | 33.33 |
| ISH1 | BIEC2_704097 | AAB | AA | BB | 261 | 2 | 1 | 66.28 | 33.33 |
| ISH1 | BIEC2_704099 | ABB | BB | AA | 261 | 2 | 1 | 66.28 | 33.33 |
| ISH1 | BIEC2_704380 | ABB | BB | AA | 261 | 2 | 1 | 66.28 | 33.33 |
| ISH1 | BIEC2_704399 | ABB | BB | AA | 261 | 2 | 1 | 66.28 | 33.33 |
| ISH1 | BIEC2_704405 | AAB | AA | BB | 261 | 2 | 1 | 66.28 | 33.33 |
| ISH1 | BIEC2_704418 | ABB | BB | AA | 261 | 2 | 1 | 66.28 | 33.33 |
| ISH2 | BIEC2_704461 | AAB | BB | AA | 306 | 1 | 2 | 33.66 | 66.01 |
| ISH2 | BIEC2_704653 | ABB | AA | BB | 306 | 1 | 2 | 33.66 | 66.01 |
| ISH1 | BIEC2_704653 | ABB | BB | AA | 261 | 2 | 1 | 66.28 | 33.33 |
| ISH1 | BIEC2_704777 | ABB | BB | AA | 261 | 2 | 1 | 66.28 | 33.33 |
| ISH1 | BIEC2_705103 | ABB | BB | AA | 261 | 2 | 1 | 66.28 | 33.33 |
| ISH2 | BIEC2_705197 | ABB | AA | BB | 306 | 1 | 2 | 33.66 | 66.01 |
| ISH1 | BIEC2_706127 | AAB | AA | BB | 261 | 2 | 1 | 66.28 | 33.33 |
| ISH1 | BIEC2_706177 | AAB | AA | BB | 261 | 2 | 1 | 66.28 | 33.33 |
| ISH2 | BIEC2_706223 | ABB | AA | BB | 306 | 1 | 2 | 33.66 | 66.01 |
| ISH2 | BIEC2_706224 | ABB | AA | BB | 306 | 1 | 2 | 33.66 | 66.01 |

| | | | | | | | | | |
|---|---|---|---|---|---|---|---|---|---|
| ISH2 | BIEC2_706481 | AAB | BB | AA | 306 | 1 | 2 | 33.66 | 66.01 |
| ISH1 | BIEC2_706573 | AAB | AA | BB | 261 | 2 | 1 | 66.28 | 33.33 |
| ISH2 | BIEC2_707219 | ABB | AA | BB | 306 | 1 | 2 | 33.66 | 66.01 |
| ISH2 | BIEC2_707260 | ABB | AA | BB | 306 | 1 | 2 | 33.66 | 66.01 |
| ISH2 | BIEC2_707269 | ABB | AA | BB | 306 | 1 | 2 | 33.66 | 66.01 |
| ISH2 | BIEC2_707307 | ABB | AA | BB | 306 | 1 | 2 | 33.66 | 66.01 |
| ISH2 | BIEC2_707341 | ABB | AA | BB | 306 | 1 | 2 | 33.66 | 66.01 |
| ISH2 | BIEC2_707446 | AAB | BB | AA | 306 | 1 | 2 | 33.66 | 66.01 |
| ISH1 | BIEC2_707925 | ABB | BB | AA | 261 | 2 | 1 | 66.28 | 33.33 |
| ISH1 | BIEC2_707930 | AAB | AA | BB | 261 | 2 | 1 | 66.28 | 33.33 |
| ISH1 | BIEC2_707945 | AAB | AA | BB | 261 | 2 | 1 | 66.28 | 33.33 |
| ISH1 | BIEC2_708018_ilmndup1 | AAB | AA | BB | 261 | 2 | 1 | 66.28 | 33.33 |
| ISH1 | BIEC2_708036 | BBB | BB | AA | 261 | 2 | 0 | 66.28 | 33.33 |
| ISH1 | BIEC2_708100 | AAB | AA | BB | 261 | 2 | 1 | 66.28 | 33.33 |
| ISH1 | BIEC2_708101 | ABB | BB | AA | 261 | 2 | 1 | 66.28 | 33.33 |
| ISH1 | BIEC2_708208 | ABB | BB | AA | 261 | 2 | 1 | 66.28 | 33.33 |
| ISH2 | BIEC2_709887 | AAB | BB | AA | 306 | 1 | 2 | 33.66 | 66.01 |
| ISH2 | BIEC2_710167 | AAB | AA | BB | 306 | 2 | 1 | 33.66 | 66.01 |
| ISH2 | BIEC2_710195 | ABB | AA | BB | 306 | 1 | 2 | 33.66 | 66.01 |
| ISH2 | BIEC2_710452 | ABB | AA | BB | 306 | 1 | 2 | 33.66 | 66.01 |
| ISH1 | BIEC2_711100 | AAB | AA | BB | 261 | 2 | 1 | 66.28 | 33.33 |
| ISH1 | BIEC2_711102 | ABB | BB | AA | 261 | 2 | 1 | 66.28 | 33.33 |
| ISH1 | BIEC2_711305 | AAB | AA | BB | 261 | 2 | 1 | 66.28 | 33.33 |
| ISH2 | BIEC2_711414 | AAB | BB | AA | 306 | 1 | 2 | 33.66 | 66.01 |
| ISH1 | BIEC2_712675 | ABB | BB | AA | 261 | 2 | 1 | 66.28 | 33.33 |
| ISH1 | BIEC2_712694 | ABB | AA | BB | 261 | 1 | 2 | 66.28 | 33.33 |
| ISH2 | BIEC2_713320 | AAB | BB | AA | 306 | 1 | 2 | 33.66 | 66.01 |
| ISH2 | BIEC2_715111 | AAB | BB | AA | 306 | 1 | 2 | 33.66 | 66.01 |
| ISH2 | BIEC2_715143 | AAB | BB | AA | 306 | 1 | 2 | 33.66 | 66.01 |
| ISH2 | BIEC2_715159 | ABB | AA | BB | 306 | 1 | 2 | 33.66 | 66.01 |
| ISH2 | BIEC2_715203 | AAB | BB | AA | 306 | 1 | 2 | 33.66 | 66.01 |

| | | | | | | | | | |
|---|---|---|---|---|---|---|---|---|---|
| ISH2 | BIEC2_715282 | AAB | BB | AA | 306 | 1 | 2 | 33.66 | 66.01 |
| ISH1 | BIEC2_715282 | ABB | BB | AA | 261 | 2 | 1 | 66.28 | 33.33 |
| ISH2 | BIEC2_715433 | ABB | AA | BB | 306 | 1 | 2 | 33.66 | 66.01 |
| ISH1 | BIEC2_715580 | ABB | BB | AA | 261 | 2 | 1 | 66.28 | 33.33 |
| ISH2 | BIEC2_715638 | ABB | AA | BB | 306 | 1 | 2 | 33.66 | 66.01 |
| ISH2 | BIEC2_715692 | AAB | BB | AA | 306 | 1 | 2 | 33.66 | 66.01 |
| ISH1 | BIEC2_715692 | ABB | BB | AA | 261 | 2 | 1 | 66.28 | 33.33 |
| ISH1 | BIEC2_715731 | AAB | AA | BB | 261 | 2 | 1 | 66.28 | 33.33 |
| ISH2 | BIEC2_716078 | AAB | BB | AA | 306 | 1 | 2 | 33.66 | 66.01 |
| ISH2 | BIEC2_716079 | ABB | AA | BB | 306 | 1 | 2 | 33.66 | 66.01 |
| ISH2 | BIEC2_716391 | ABB | AA | BB | 306 | 1 | 2 | 33.66 | 66.01 |
| ISH2 | BIEC2_718377 | AAB | BB | AA | 306 | 1 | 2 | 33.66 | 66.01 |
| ISH2 | BIEC2_718915 | AAB | BB | AA | 306 | 1 | 2 | 33.66 | 66.01 |
| ISH2 | BIEC2_719149 | AAB | BB | AA | 306 | 1 | 2 | 33.66 | 66.01 |
| ISH2 | BIEC2_719698 | AAB | BB | AA | 306 | 1 | 2 | 33.66 | 66.01 |
| ISH2 | BIEC2_720497 | AAB | BB | AA | 306 | 1 | 2 | 33.66 | 66.01 |
| ISH2 | BIEC2_720856 | ABB | AA | BB | 306 | 1 | 2 | 33.66 | 66.01 |
| ISH1 | BIEC2_720856 | ABB | BB | AA | 261 | 2 | 1 | 66.28 | 33.33 |
| ISH2 | BIEC2_721123 | ABB | AA | BB | 306 | 1 | 2 | 33.66 | 66.01 |
| ISH2 | BIEC2_721407 | ABB | AA | BB | 306 | 1 | 2 | 33.66 | 66.01 |
| ISH1 | BIEC2_721407 | ABB | BB | AA | 261 | 2 | 1 | 66.28 | 33.33 |
| ISH2 | BIEC2_721414 | ABB | AA | BB | 306 | 1 | 2 | 33.66 | 66.01 |
| ISH1 | BIEC2_721414 | ABB | BB | AA | 261 | 2 | 1 | 66.28 | 33.33 |
| ISH2 | BIEC2_721927 | AAB | BB | AA | 306 | 1 | 2 | 33.66 | 66.01 |
| ISH2 | BIEC2_722004 | AAB | BB | AA | 306 | 1 | 2 | 33.66 | 66.01 |
| ISH2 | BIEC2_722051 | ABB | AA | BB | 306 | 1 | 2 | 33.66 | 66.01 |
| ISH2 | BIEC2_722209 | ABB | AA | BB | 306 | 1 | 2 | 33.66 | 66.01 |
| ISH2 | BIEC2_722308 | AAB | BB | AA | 306 | 1 | 2 | 33.66 | 66.01 |
| ISH1 | BIEC2_722463 | ABB | BB | AA | 261 | 2 | 1 | 66.28 | 33.33 |
| ISH2 | BIEC2_722604 | ABB | AA | BB | 306 | 1 | 2 | 33.66 | 66.01 |
| ISH2 | BIEC2_722966 | AAB | BB | AA | 306 | 1 | 2 | 33.66 | 66.01 |

| | | | | | | | | | |
|---|---|---|---|---|---|---|---|---|---|
| ISH1 | BIEC2_735411 | ABB | BB | AA | 261 | 2 | 1 | 66.28 | 33.33 |
| ISH1 | BIEC2_735928 | AAB | AA | BB | 261 | 2 | 1 | 66.28 | 33.33 |
| ISH1 | BIEC2_737243 | AAB | AA | BB | 261 | 2 | 1 | 66.28 | 33.33 |
| ISH1 | BIEC2_739123 | ABB | BB | AA | 261 | 2 | 1 | 66.28 | 33.33 |
| ISH1 | BIEC2_739125 | AAB | AA | BB | 261 | 2 | 1 | 66.28 | 33.33 |
| ISH2 | BIEC2_739516 | AAB | BB | AA | 306 | 1 | 2 | 33.66 | 66.01 |
| ISH1 | BIEC2_740399 | ABB | BB | AA | 261 | 2 | 1 | 66.28 | 33.33 |
| ISH2 | BIEC2_740857 | AAB | BB | AA | 306 | 1 | 2 | 33.66 | 66.01 |
| ISH2 | BIEC2_741890 | AAB | BB | AA | 306 | 1 | 2 | 33.66 | 66.01 |
| ISH2 | BIEC2_741900 | ABB | AA | BB | 306 | 1 | 2 | 33.66 | 66.01 |
| ISH2 | BIEC2_741929 | BBB | AA | BB | 306 | 0 | 2 | 33.66 | 66.01 |
| ISH1 | BIEC2_742363 | AAB | AA | BB | 261 | 2 | 1 | 66.28 | 33.33 |
| ISH1 | BIEC2_742533 | ABB | BB | AA | 261 | 2 | 1 | 66.28 | 33.33 |
| ISH1 | BIEC2_742729 | AAB | AA | BB | 261 | 2 | 1 | 66.28 | 33.33 |
| ISH1 | BIEC2_742760 | ABB | BB | AA | 261 | 2 | 1 | 66.28 | 33.33 |
| ISH2 | BIEC2_743286 | AAB | BB | AA | 306 | 1 | 2 | 33.66 | 66.01 |
| ISH1 | BIEC2_743663 | ABB | BB | AA | 261 | 2 | 1 | 66.28 | 33.33 |
| ISH1 | BIEC2_743709 | AAB | AA | BB | 261 | 2 | 1 | 66.28 | 33.33 |
| ISH2 | BIEC2_743824 | ABB | AA | BB | 306 | 1 | 2 | 33.66 | 66.01 |
| ISH2 | BIEC2_744129 | AAB | BB | AA | 306 | 1 | 2 | 33.66 | 66.01 |
| ISH2 | BIEC2_745270 | ABB | AA | BB | 306 | 1 | 2 | 33.66 | 66.01 |
| ISH1 | BIEC2_747061 | AAB | AA | BB | 261 | 2 | 1 | 66.28 | 33.33 |
| ISH2 | BIEC2_748314 | ABB | AA | BB | 306 | 1 | 2 | 33.66 | 66.01 |
| ISH1 | BIEC2_748314 | ABB | BB | AA | 261 | 2 | 1 | 66.28 | 33.33 |
| ISH2 | BIEC2_749242 | ABB | AA | BB | 306 | 1 | 2 | 33.66 | 66.01 |
| ISH1 | BIEC2_749740 | ABB | BB | AA | 261 | 2 | 1 | 66.28 | 33.33 |
| ISH2 | BIEC2_751550 | AAB | BB | AA | 306 | 1 | 2 | 33.66 | 66.01 |
| ISH2 | BIEC2_752280 | ABB | AA | BB | 306 | 1 | 2 | 33.66 | 66.01 |
| ISH2 | BIEC2_752687 | AAB | BB | AA | 306 | 1 | 2 | 33.66 | 66.01 |
| ISH2 | BIEC2_756150 | ABB | AA | BB | 306 | 1 | 2 | 33.66 | 66.01 |
| ISH2 | BIEC2_756191 | ABB | AA | BB | 306 | 1 | 2 | 33.66 | 66.01 |

| | | | | | | | | | |
|---|---|---|---|---|---|---|---|---|---|
| ISH2 | BIEC2_757190 | ABB | AA | BB | 306 | 1 | 2 | 33.66 | 66.01 |
| ISH2 | BIEC2_757254 | AAB | BB | AA | 306 | 1 | 2 | 33.66 | 66.01 |
| ISH2 | BIEC2_759331 | ABB | AA | BB | 306 | 1 | 2 | 33.66 | 66.01 |
| ISH2 | TBIEC2_740109 | ABB | AA | BB | 306 | 1 | 2 | 33.66 | 66.01 |
| ISH2 | TBIEC2_741072 | AAB | BB | AA | 306 | 1 | 2 | 33.66 | 66.01 |
| ISH2 | TBIEC2_742809 | ABB | AA | BB | 306 | 1 | 2 | 33.66 | 66.01 |
| ISH2 | TBIEC2_742971 | AAB | BB | AA | 306 | 1 | 2 | 33.66 | 66.01 |
| ISH2 | TBIEC2_742975 | ABB | AA | BB | 306 | 1 | 2 | 33.66 | 66.01 |
| ISH2 | UKUL3955 | AAB | BB | AA | 306 | 1 | 2 | 33.66 | 66.01 |
| ISH2 | UKUL3961 | ABB | AA | BB | 306 | 1 | 2 | 33.66 | 66.01 |
| ISH2 | UKUL3993 | AAB | BB | AA | 306 | 1 | 2 | 33.66 | 66.01 |

**Supplementary Table S2.** Haematology and biochemistry test results for the ISH1 colt.

| Test | Units | Result | Reference Range | Explanation |
|---|---|---|---|---|
| RBC (Red Blood Cell count) | $10^{12}$/L | 7.03 | (9.10 - 10.70) | Red Blood Cell count – measures the number of RBCs in the blood. |
| PCV (Packed Cell Volume or Hematocrit) | L/L | 30.5 | (38.80 - 44.80) | The proportion of blood volume occupied by red blood cells. |
| Hb (Haemoglobin) | g/dL | 11 | (12.50 - 15.30) | The amount of haemoglobin (oxygen-carrying protein) in the blood. |
| MCV (Mean Corpuscular Volume) | fL | 43.5 | (41.00 - 43.80) | The average size of red blood cells. |
| MCHC (Mean Corpuscular Haemoglobin Concentration) | g/dL | 36 | (34.00 - 35.20) | The concentration of haemoglobin in red blood cells. |
| MCH (Mean Corpuscular Haemoglobin) | pg | 15.7 | (14.20 - 15.20) | The average amount of haemoglobin per red blood cell. |
| Plts (Platelets) | $10^9$/L | 145 | (141.00 - 191.00) | The number of platelets, which help in blood clotting. |
| WBC (White Blood Cell Count) | $10^9$/L | 10.7 | (7.60 - 9.40) | The number of white blood cells, indicating immune response. |
| Neuts (Neutrophils) | % / $10^9$/L | 49% / 5.24 | (53.00 - 63.00) | First-line defense against infections. |
| Lymphs (Lymphocytes) | % / $10^9$/L | 44% / 4.71 | (31.00 - 49.00) | Essential for immune function and antibody production. |
| Monos (Monocytes) | % / $10^9$/L | 5% / 0.53 | (5.00 - 7.00) | Help fight infections and clear dead cells. |
| Eos (Eosinophils) | % / $10^9$/L | 2% / 0.21 | (0.60 - 1.40) | Involved in allergic reactions and parasitic infections. |
| T. Prot (Total Protein) | g/L | 59.4 | (54.20 - 59.00) | Measures all proteins in blood, including albumin and globulins. |
| Alb (Albumin) | g/L | 30.9 | (32.70 - 35.30) | A protein that maintains blood volume and transports substances. |

| | | | | |
|---|---|---|---|---|
| Glob (Globulin) | g/L | 28.5 | (20.50 - 30.00) | Includes antibodies and other immune proteins. |
| AST (Aspartate Aminotransferase) | IU/L | 317 | (327.00 - 539.00) | An enzyme found in the liver and muscles, indicating liver/muscle health. |
| CK (Creatine Kinase) | IU/L | 343 | (160.00 - 240.00) | An enzyme linked to muscle damage. |
| GGT (Gamma-Glutamyl Transferase) | IU/L | 10.1 | (16.00 - 35.20) | An enzyme related to liver function. |
| T. Bili (Total Bilirubin) | µmol/L | 10.6 | (36.70 - 57.36) | A waste product from red blood cell breakdown, indicating liver function. |
| Na (Sodium) | mmol/L | 137 | (137.00 - 141.00) | An electrolyte involved in fluid balance and nerve function. |
| K (Potassium) | mmol/L | 4.53 | (3.40 - 4.00) | Essential for muscle and nerve function. |
| Cl (Chloride) | mmol/L | 100 | (99.00 - 103.00) | Helps maintain blood acidity and hydration. |
| Iron | µmol/L | 33.2 | (24.30 - 36.90) | Required for haemoglobin production. |
| Urea | mmol/L | 7.1 | (<8.00) | Indicates kidney function and protein metabolism. |
| Creat (Creatinine) | µmol/L | 103 | (<180.00) | A waste product from muscle metabolism, assessing kidney function. |
| Bile Acids | µmol/L | 2.6 | N/A | Indicates liver function and bile acid metabolism. |

**Supplementary Table S3.** The length and the number of genes on each bovine autosome according to the ARS-UCD 1.2 genome build.

| Autosome | Length of autosome [mp] | Number of genes | Gene density |
|---|---|---|---|
| 1 | 158.5 | 1267 | 8.0 |
| 2 | 136.2 | 1237 | 9.1 |
| 3 | 121.0 | 1832 | 15.1 |
| 4 | 120.0 | 1189 | 9.9 |
| 5 | 120.1 | 1716 | 14.3 |
| 6 | 117.8 | 953 | 8.1 |
| 7 | 110.7 | 1816 | 16.4 |
| 8 | 113.3 | 1099 | 9.7 |
| 9 | 105.5 | 822 | 7.8 |
| 10 | 103.3 | 1567 | 15.2 |
| 11 | 107.0 | 1311 | 12.3 |
| 12 | 87.2 | 660 | 7.6 |
| 13 | 83.5 | 1111 | 13.3 |
| 14 | 82.4 | 734 | 8.9 |
| 15 | 85.0 | 1491 | 17.5 |
| 16 | 81.0 | 948 | 11.7 |
| 17 | 73.2 | 905 | 12.4 |
| 18 | 65.8 | 1648 | 25.0 |
| 19 | 63.4 | 1570 | 24.7 |
| 20 | 72.0 | 510 | 7.1 |
| 21 | 69.9 | 859 | 12.3 |
| 22 | 60.8 | 762 | 12.5 |
| 23 | 52.5 | 1134 | 21.6 |
| 24 | 62.3 | 469 | 7.5 |
| 25 | 42.4 | 973 | 23.0 |
| 26 | 52.0 | 559 | 10.8 |
| 27 | 45.6 | 377 | 8.3 |
| 28 | 45.9 | 444 | 9.7 |
| 29 | 51.1 | 903 | 17.7 |

Ensembl: http://www.ensembl.org/index.html for assembly size and annotated gene content.

**Supplementary Table S4.** The length and the number of genes on each equine autosome according to the EquCab3.0. adapted from Raudsepp, *et al.* [48].

| Autosome | Length of autosome [mp] | Number of genes | Gene density |
|---|---|---|---|
| 1 | 188.3 | 2388 | 12.7 |
| 2 | 121.4 | 1527 | 12.6 |
| 3 | 121.4 | 1274 | 10.5 |
| 4 | 109.5 | 1089 | 9.9 |
| 5 | 96.8 | 1321 | 13.6 |
| 6 | 87.2 | 1314 | 15.1 |
| 7 | 100.8 | 1753 | 17.4 |
| 8 | 97.6 | 1170 | 12.0 |
| 9 | 85.8 | 743 | 8.7 |
| 10 | 85.2 | 1492 | 17.5 |
| 11 | 61.7 | 1423 | 23.1 |
| 12 | 37 | 915 | 24.7 |
| 13 | 43.8 | 886 | 20.2 |
| 14 | 94.6 | 1061 | 11.2 |
| 15 | 92.9 | 1040 | 11.2 |
| 16 | 89 | 1012 | 11.4 |
| 17 | 80.7 | 626 | 7.8 |
| 18 | 82.6 | 675 | 8.2 |
| 19 | 62.7 | 626 | 10.0 |
| 20 | 65.3 | 1000 | 15.3 |
| 21 | 59 | 587 | 9.9 |
| 22 | 50.9 | 804 | 15.8 |
| 23 | 55.6 | 545 | 9.8 |
| 24 | 48.3 | 751 | 15.5 |
| 25 | 40.3 | 714 | 17.7 |
| 26 | 43.1 | 361 | 8.4 |
| 27 | 40.3 | 342 | 8.5 |
| 28 | 47.3 | 577 | 12.2 |
| 29 | 34.8 | 326 | 9.4 |
| 30 | 31.4 | 289 | 9.2 |
| 31 | 26 | 240 | 9.2 |
